# Nitric Oxide Induces Cell Death by Regulating Anti-Apoptotic BCL-2 Family Members

**DOI:** 10.1371/journal.pone.0007059

**Published:** 2009-09-21

**Authors:** Colleen M. Snyder, Emelyn H. Shroff, Jing Liu, Navdeep S. Chandel

**Affiliations:** 1 Department of Medicine, Northwestern University Medical School, Chicago, Illinois, United States of America; 2 Department of Cell and Molecular Biology, Northwestern University Medical School, Chicago, Illinois, United States of America; Health Canada, Canada

## Abstract

Nitric oxide (NO) activates the intrinsic apoptotic pathway to induce cell death. However, the mechanism by which this pathway is activated in cells exposed to NO is not known. Here we report that BAX and BAK are activated by NO and that cytochrome c is released from the mitochondria. Cells deficient in *Bax* and *Bak* or Caspase-9 are completely protected from NO-induced cell death. The individual loss of the BH3-only proteins, *Bim*, *Bid*, *Puma*, *Bad* or *Noxa*, or *Bid* knockdown in *Bim^−/−^/Puma^−/−^* MEFs, does not prevent NO-induced cell death. Our data show that the anti-apoptotic protein MCL-1 undergoes ASK1-JNK1 mediated degradation upon exposure to NO, and that cells deficient in either *Ask1* or *Jnk1* are protected against NO-induced cell death. NO can inhibit the mitochondrial electron transport chain resulting in an increase in superoxide generation and peroxynitrite formation. However, scavengers of ROS or peroxynitrite do not prevent NO-induced cell death. Collectively, these data indicate that NO degrades MCL-1 through the ASK1-JNK1 axis to induce BAX/BAK-dependent cell death.

## Introduction

Nitric oxide (NO) has been shown to have both protective and deleterious functions. This molecule is critical for multiple physiological functions, yet plays a role in many pathological states, including neurodegenerative disease and cancer. A major target of NO is cytochrome oxidase (COX), the terminal enzyme of the electron transport chain (ETC) [1]–[3]. NO binds COX reversibly to regulate cellular respiration. Although NO's ability to modify biological molecules has proven to be physiologically important, NO-dependent modifications may contribute to cell death through S-nitrosylation of proteins or the formation of peroxynitrite. Prolonged exposure to NO inhibits complex I of the ETC likely through *S*-nitrosylation [4]. Inhibition of complex I by NO has been hypothesized to result in an elevated release of oxidants from the ETC [5]. An increase in oxidants in the presence of NO favors the formation of the highly reactive molecule peroxynitrite which can activate p38 and c-Jun N-terminal kinase (JNK) to initiate the intrinsic apoptotic pathway [6]–[8].

The BCL-2 family of proteins regulate the intrinsic apoptotic pathway. These proteins can be divided into two subclasses, the anti-apoptotic proteins (BCL-2, BCL-X_L_, BCL-w, MCL-1 and A1), and the pro-apoptotic proteins which include the multi-domain BCL-2 proteins (BAX, BAK and BOK) and the BH3-only proteins (including BIM, BID, PUMA, BAD and NOXA). In healthy cells, BAX and BAK exist as monomers in the cytosol and at the mitochondrial outer membrane, respectively [9]. In response to a death stimulus, the BH3-only proteins are activated transcriptionally and/or by post-translational modifications and cause the oligomerization of BAX and BAK resulting in mitochondrial outer membrane permeabilization (MOMP). This allows proteins that normally reside in the intermembrane space, such as cytochrome c, to enter the cytosol [10], [11]. When cytochrome c is released into the cytosol, it forms a complex with APAF-1 and pro-caspase-9 in an ATP-dependent manner resulting in the formation of the apoptosome which in turn activates downstream executioner caspases resulting in apoptosis [12], [13].

BH3-only proteins and the anti-apoptotic proteins are upstream regulators of BAX and BAK activation yet the exact mechanism by which these proteins initiate apoptosis is not completely understood. Currently, there are two models that describe how these proteins mediate BAX and BAK activity, the indirect activation model and the direct activation model. The indirect activation model postulates that in healthy cells, anti-apoptotic proteins associate with BAX and BAK and repress their activity [14]. In response to a death stimulus, BH3-only proteins interact with the anti-apoptotic proteins, displacing them from BAX and BAK resulting in MOMP. This model assumes that BAX and BAK are kept in check by anti-apoptotic proteins. The direct activation model divides the BH3-only proteins into “activators” (BID, BIM and PUMA) and “sensitizers” (all other BH3-only proteins). This model proposes that the anti-apoptotic family members prevent cell death by disrupting signaling upstream of BAX and BAK by binding to the “activator” BH3-only proteins and negating their function [15]. In response to a death stimulus, the “sensitizer” BH3-only proteins bind to the anti-apoptotic proteins, releasing the “activators” and allowing them to induce apoptosis by directly activating BAX and BAK. Despite suggesting different mechanisms of action, both models agree that the anti-apoptotic BCL-2 proteins need to be negated for BAX and BAK mediated MOMP.

Previous studies have demonstrated that the loss of BAX and BAK, prevents NO induction of cell death [16]–[19]. As mentioned, BAX and BAK activation is regulated by BH3-only proteins yet it is not known which BH3-only proteins are involved in NO-induced cell death. Furthermore, the signaling pathways that are initiated by NO to activate BAX/BAK-induced cell death are also unknown. In the present study, we investigated which signaling pathways and BH3-only proteins cause the negation of anti-apoptotic BCL-2 proteins to cause NO-induced cell death.

## Results

### Nitric oxide-induced cell death requires the intrinsic apoptotic proteins, BAX, BAK and Caspase-9, and causes release of cytochrome c from the mitochondria

**Figure 1:**
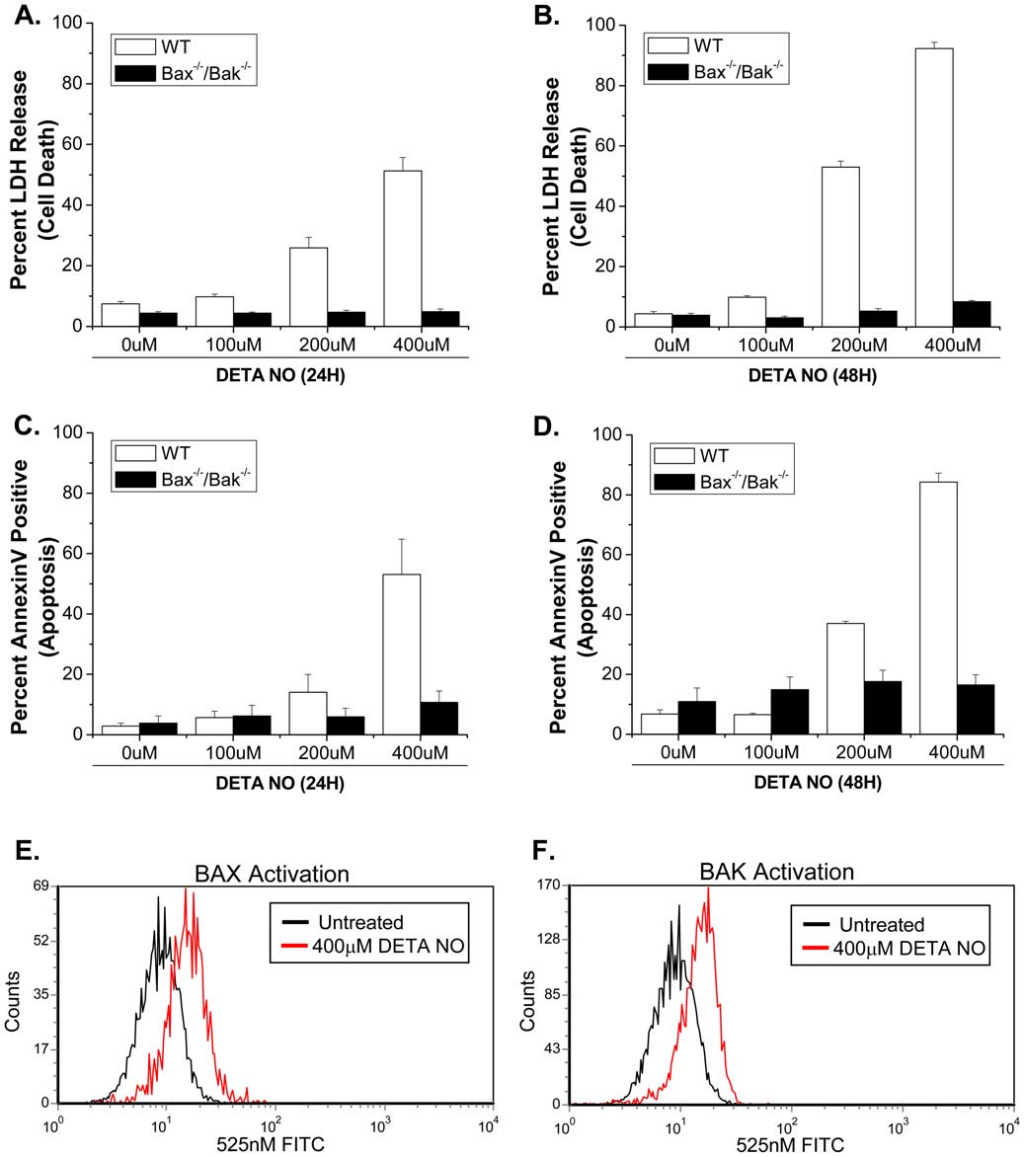
Nitric oxide induces apoptosis through the intrinsic apoptotic pathway. Wild type and *Bax^−/−^/Bak^−/−^* MEFs were treated with 0, 100, 200 and 400 µM DETA-NO for 24 (A/C) and 48 (B/D) hours. Cell death was measured by percent LDH release (A/B). Apoptosis was measured by percent Annexin V and propidium iodide staining (C/D). BAX and BAK activation was determined in wild type MEFs treated with (red) and without (black) DETA-NO (400 µM) for 24 hours (E/F).

**Figure 2:**
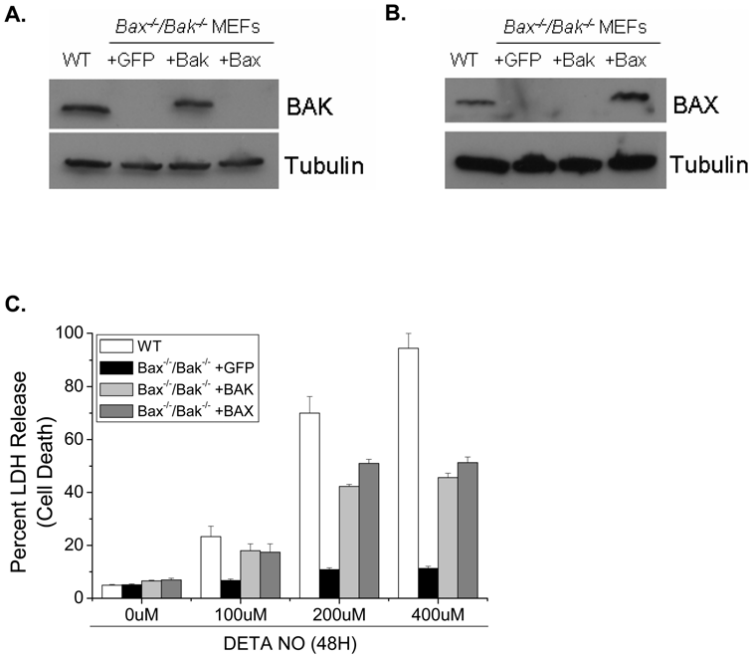
BAX and BAK mediate nitric oxide-indeced cell death. *Bax^−/−^/Bak^−/−^* MEFs were infected with either Bax, Bak or GFP as a control. BAX and BAK expression was verified by Western analysis (A,B). *Bax^−/−^/Bak^−/−^* MEFs expressing GFP, BAK or BAX were treated with 0, 100, 200 and 400 µM DETA-NO for 48 hours and cell death was measured by LDH release (C).

**Figure 3:**
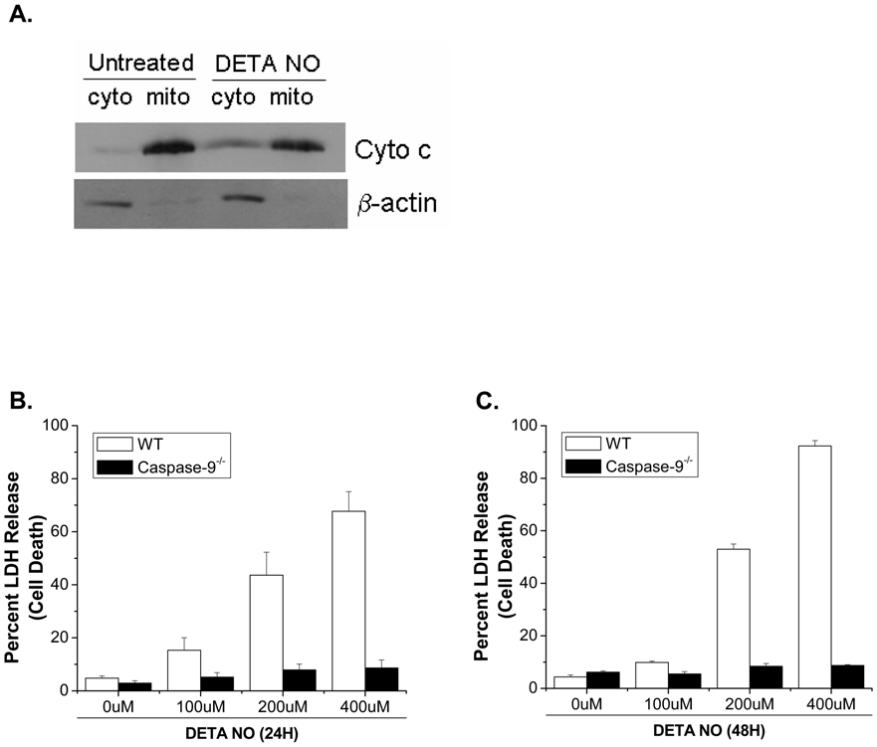
Cytochrome c is released and caspase-9 is required for nitric oxide-induced cell death. Cytochrome c release was measured in wild type MEFs treated with 400 µM DETA-NO for 24 hours (A). β-actin was used as a loading control. Wild type and Caspase-9^−/−^ MEFs were treated with 0, 100, 200 and 400 µM DETA-NO for 24 (B) and 48 hours (C) and cell death was measured by LDH release.

To investigate the mechanism by which long-term exposure to NO cause's cell death we used the slow-releasing NO donor, diethylenetriamine (DETA)-NO. DETA-NO mimic's steady state production of NO at concentrations observed with activated macrophages [24], [25]. BAX, BAK and Caspase-9 are integral members of the intrinsic apoptotic pathway. To determine if NO induces cell death in a BAX/BAK-dependent manner, wild type and *Bax^−/−^/Bak^−/−^* MEFs were exposed to DETA-NO for 24 and 48 hours. Cell death was measured by LDH release, a pan marker of plasma membrane disruption. Wild type MEFs show a concentration dependent increase in cell death in response to DETA-NO whereas, *Bax^−/−^/Bak^−/−^* MEFs are completely protected at all concentrations and time points (Figure 1A/B). To confirm that NO induces BAX/BAK-dependent apoptosis, wild type and *Bax^−/−^/Bak^−/−^* MEFs were treated with DETA-NO and stained with annexin V. Wild type MEFs show increased apoptosis in response to NO, whereas *Bax^−/−^/Bak^−/−^* MEFs are completely protected (Figure 1C/D). To determine if BAX and BAK are activated in response to NO, wild type MEFs were treated with DETA-NO for 24 hours and BAX and BAK activation was determined using antibodies that specifically recognize the activated form of these proteins [20], [23]. Indeed, BAX and BAK are activated in response to NO treatment indicating that both of these proteins regulate NO-mediated apoptosis (Figure 1E/F). *Bax* or *Bak* reconstitution sensitized *Bax^−/−^/Bak^−/−^* MEFs to NO-induced apoptosis indicating that these proteins, individually, are involved in this pathway (Figure 2A–C). Cytochrome c release and caspase-9 activation occur downstream of BAX and BAK activation and are required for BAX/BAK-dependent apoptosis. Cytochrome c is released in wild-type MEFs exposed to DETA-NO (Figure 3A). Additionally, *Caspase-9^−/−^* MEFs treated with DETA-NO for 24 and 48 hours did not undergo cell death (Figure 3B, C).

### The individual loss of the BH3-only proteins, BID, BIM, PUMA, BAD or NOXA, is not sufficient to protect against nitric oxide-induced cell death

**Figure 4:**
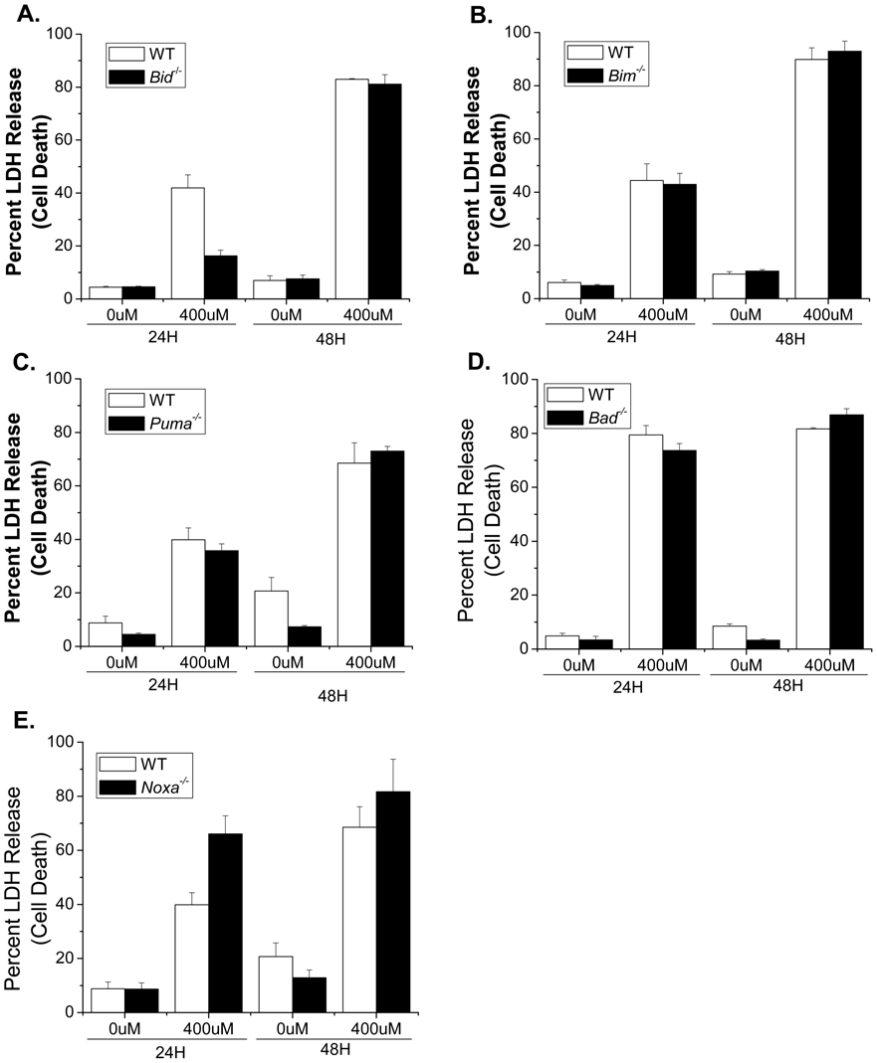
The individual loss of the BH3-only proteins BID, BIM, PUMA, BAD and NOXA does not protect against nitric oxide-induced cell death. Wild type, *Bid^−/−^* (A), *Bim^−/−^* (B), *Puma^−/−^* (C), *Bad^−/−^* (D) and *Noxa^−/−^* (E) MEFs were treated with 0 and 400 µM DETA-NO for 24 and 48 hours. Percent cell death was measured by LDH release.

The best known upstream regulators of BAX and BAK are the BH3-only proteins. Thus, we wanted to identify which BH3-only proteins activate BAX and BAK during NO-induced cell death. To test the role of individual BH3-only proteins during NO treatment, wild type, *Bid^−/−^ Bim^−/−^*, *Puma^−/−^*, *Bad^−/−^* and *Noxa^−/−^* MEFs were treated with DETA-NO for 24 and 48 hours and cell death was measured by LDH release. *Bid^−/−^* MEFs were slightly protected 24 hours after treatment compared to wild type controls but this protection was not sustained to 48 hours, indicating that the loss of *Bid* is not sufficient to protect cells from NO-induced cell death (Figure 4A). *Bim^−/−^*, *Puma^−/−^*, *Bad^−/−^* and *Noxa^−/−^* MEFs all died at rates similar to wild type controls, suggesting that these proteins are not individually required for NO-induced cell death (Figure 4B–E).

### The combined loss of direct activator BH3-only proteins does not protect cells from nitric oxide-induced cell death

**Figure 5:**
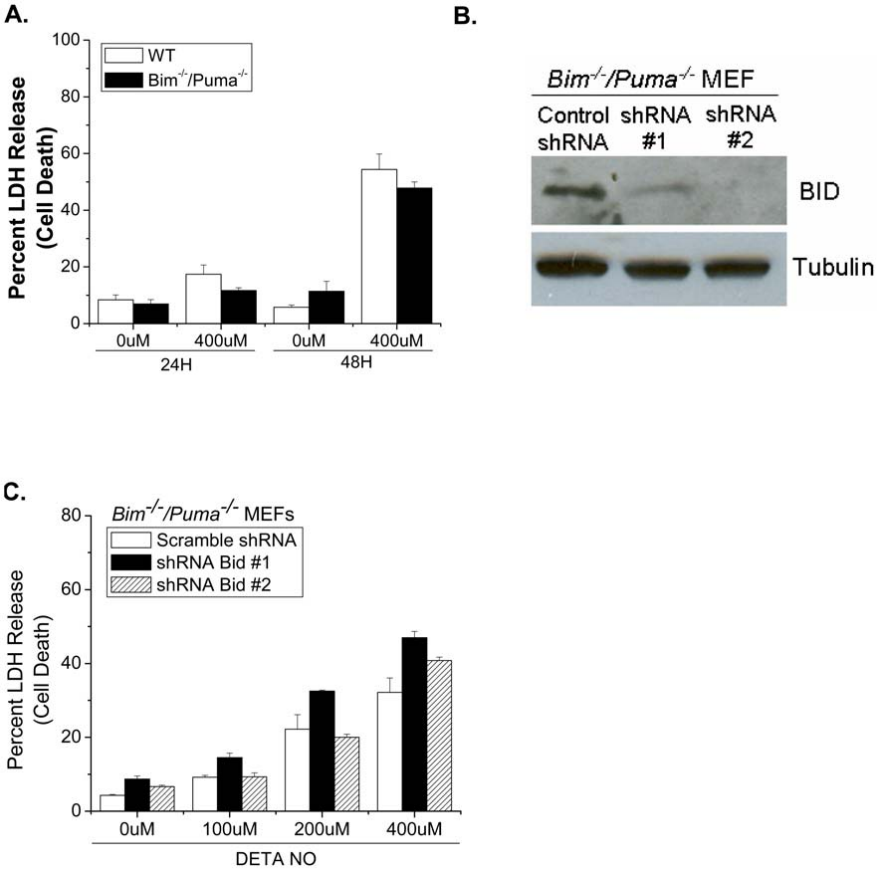
The combined loss of direct activator proteins does not protect cells from nitric oxide-induced cell death. Wild type and *Bim^−/−^/Puma^−/−^* MEFs were treated with 0 and 400 µM DETA-NO for 24 and 48 hours and cell death was measured by percent LDH release (A). BID protein expression was assessed in *Bim^−/−^/Puma^−/−^* MEFs stably expressing scrambled shRNA or shRNA targeted against *Bid* (B). *Bim^−/−^/Puma^−/−^* MEFs stably expressing scrambled shRNA or shRNA targeted against *Bid* were treated with 0, 100, 200 and 400 µM DETA-NO at the indicated concentrations for 48 hours and cell death was measured by percent LDH release (C).

The anti-apoptotic BCL-2 proteins need to be negated by the BH3-only proteins; either to release the inhibition on BAX and BAK or to release “activator” BH3-only proteins (BIM, BID, PUMA) which subsequently bind and activate BAX and BAK [14], [15]. Since the individual loss of the BH3-only proteins, BIM, BID, PUMA, BAD and NOXA did not prevent NO-induced cell death we wanted to examine if the combined loss of BH3-only proteins, namely the “activator” BH3-only proteins, protects cells from NO-induced apoptosis, wild type and *Bim^−/−^*/*Puma^−/−^* MEFs were treated with DETA-NO for 24 and 48 hours and cell death was measured by LDH release. *Bim^−/−^*/*Puma^−/−^* MEFs died at rates similar to wild type controls (Figure 5A). We reduced *Bid* expression in *Bim^−/−^*/*Puma^−/−^* MEFs using shRNA (Figure 5B). *Bim^−/−^*/*Puma^−/−^* MEFs expressing shRNA targeted against *Bid* also died at rates similar to controls (Figure 5C). These results suggest that the direct activator proteins, BIM, BID and PUMA do not regulate NO-dependent BAX/BAK activation.

### The ASK1-JNK1 axis is a key regulator in nitric oxide-induced cell death

**Figure 6:**
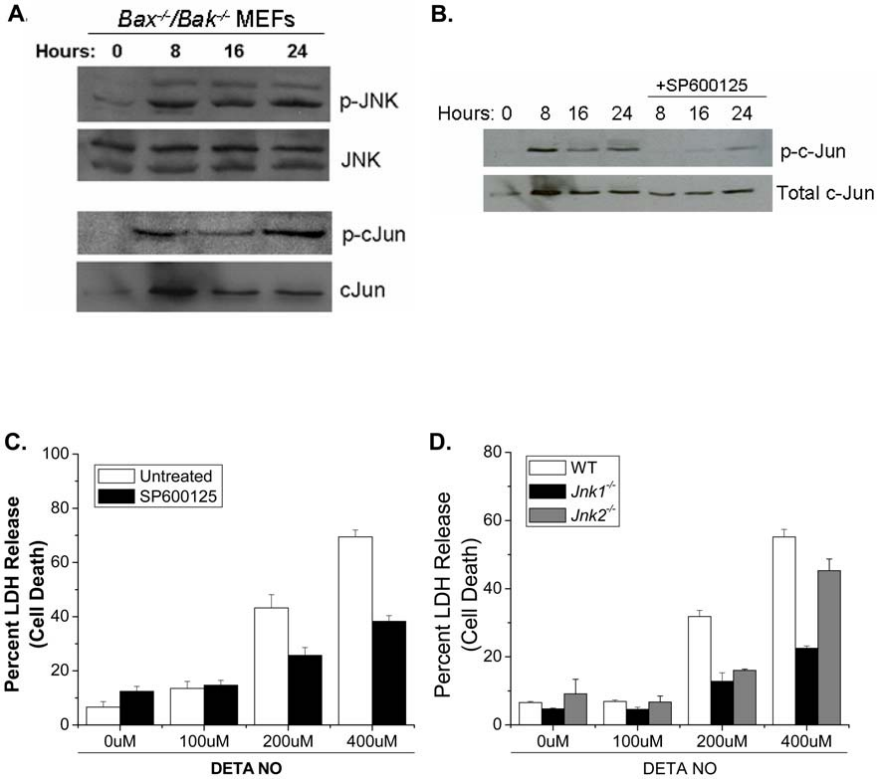
Nitric oxide initiates cell death through the ASK1-JNK1 pathway. Phospho-JNK and phospho-cJun were measured in *Bax^−/−^/Bak^−/−^* MEFs treated with 400 µM DETA-NO at 0, 8, 16 and 24 hours (A). *Bax^−/−^/Bak^−/−^* MEFs were pretreated with 40 µM SP600125 for 30 minutes followed by 400 µM DETA-NO for 8, 16 and 24 hours and phospho-c-Jun was measured (B). Wild type MEFs were pretreated with 40 µM SP600125 for 30 minutes followed by 0, 100, 200 and 400 µM DETA-NO for 24 hours. Percent cell death was measured by LDH release (C). Percent LDH release was measured in wild type, *Jnk1^−/−^* and *Jnk2^−/−^* MEFs treated with 0, 100, 200 and 400 µM DETA-NO (D).

**Figure 7:**
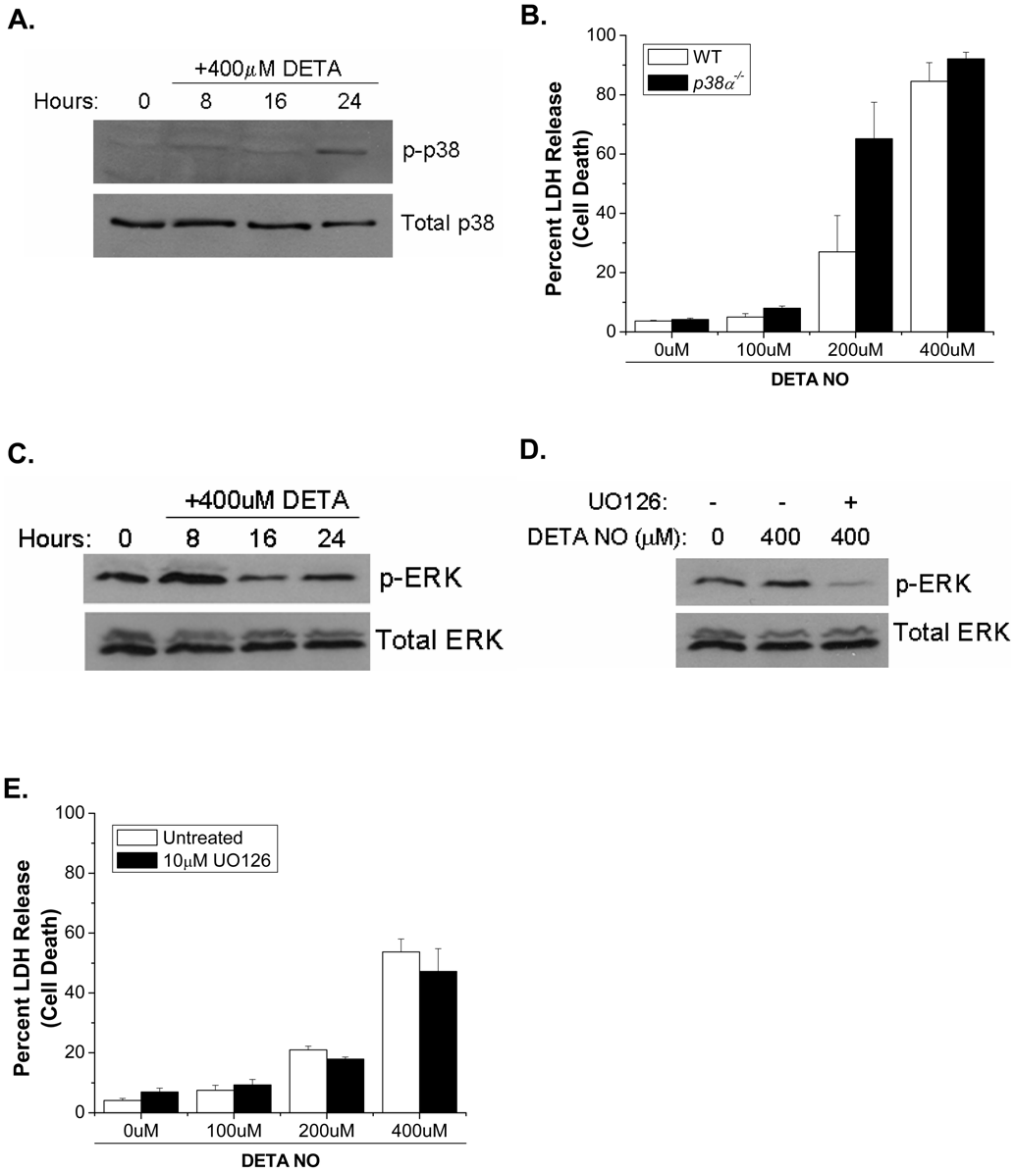
p38 and ERK are activated by NO, but do not mediate NO-induced cell death. Phospho-p38 was measured to assess p38 activity in *Bax^−/−^/Bak^−/−^* MEFs treated with 400 µM DETA-NO for 8, 16 and 24 hours (A). Percent LDH release was measured in wild type and *p38^−/−^* MEFs treated with 0, 100, 200 and 400 µM DETA-NO for 24 hours (B). Phospho-ERK was measured to assess ERK activity in *Bax^−/−^/Bak^−/−^* MEFs treated with 400 µM DETA-NO for 8, 16 and 24 hours (C). Phospho-ERK was measured in *Bax^−/−^/Bak^−/−^* MEFs pretreated with 10 µM of the ERK inhibitor UO126 followed by 400 µM DETA-NO (D). Percent LDH release was measured in wild type MEFs pretreated with UO126 (10 µM) followed by 0, 100, 200 (E).

**Figure 8:**
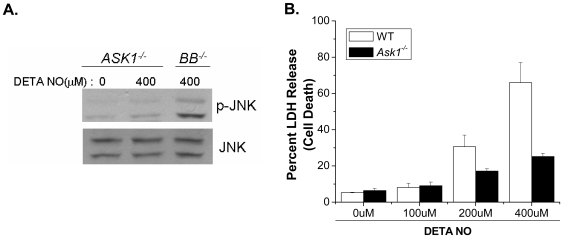
*Ask1* is required for NO-induced cell death. Phospho-JNK was measured to assess JNK activity in *Ask1^−/−^* and *Bax^−/−^/Bak^−/−^* MEFs treated with 0 or 400 µM DETA-NO (A). Percent LDH release was measured in wild type and Ask*1^−/−^* MEFs treated with 0, 100, 200 and 400 µM DETA-NO (B).

NO has been shown to modulate JNK activity [26], [27] and JNK has been shown to be an upstream regulator of BAX and BAK [28]–[30]. To determine if NO activates JNK upstream of BAX and BAK, we treated *Bax^−/−^/Bak^−/−^* MEFs with DETA-NO and assessed JNK activity by measuring expression of phoshpo-cJun, a downstream target of JNK. NO activates JNK as early as 8 hours after exposure to NO, and this activation was sustained to 24 hours (Figure 6A). Additionally, this data shows that JNK activation in response to NO occurs upstream of BAX and BAK. The JNK inhibitor SP600125 effectively inhibits NO-dependent JNK activity for up to 24 hours (Figure 6B), and chemical inhibition of JNK protects wild type MEFs from NO-induced cell death (Figure 6C). Three genes encode the JNK protein kinases. *Jnk1* and *Jnk2* are ubiquitously expressed, whereas *Jnk3* is expressed primarily in the brain, heart and testis [31]. JNK1 has been proposed as the major JNK isoform that regulates cell death [21]. Indeed, *Jnk1^−/−^* MEFs were markedly protected against NO-induced cell death compared with *Jnk2^−/−^* MEFs and wild type MEFs (Figure 6D). Interestingly, other MAPK family members such as ERK and p38 were activated by NO (Figure 7A and C) but were not required for cell death (Figures 7B and E). The apoptosis signal-regulating kinase 1 (ASK1) is regulated by NO [32]. Previous studies indicate that ASK1 is a MAPKKK that activates both JNK and p38 pathways [33]. To determine if ASK1 activates JNK in response to NO, *Ask1^−/−^* MEFs were treated with DETA-NO and phospho-JNK expression was determined by Western analysis. JNK is not phosphorylated in *Ask1^−/−^* MEFs after treatment with DETA-NO, but is phosphorylated in *Bax^−/−^/Bak^−/−^* MEFs (Figure 8A). Furthermore, *Ask1^−/−^* MEFs treated with DETA-NO are protected from NO-induced cell death as measured by LDH release (Figure 8B). These results indicate that NO activates ASK1 which subsequently initiates JNK1-dependent cell death.

### Nitric oxide induces ASK1-JNK1-dependent degradation of MCL-1

**Figure 9:**
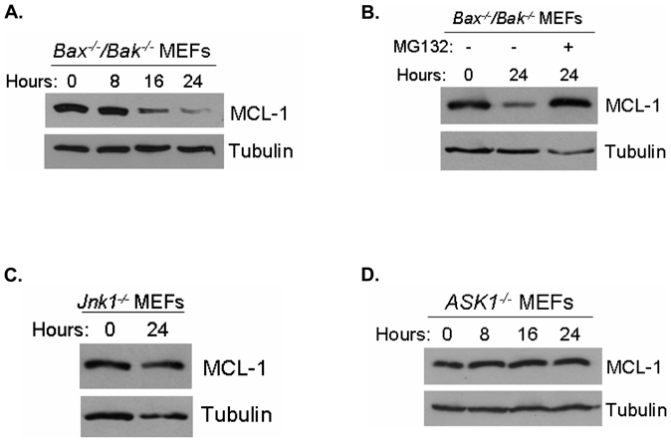
MCL-1 is degraded in response to nitric oxide treatment and is dependent on the ASK1/JNK1 pathway. MCL-1 protein expression was measured in *Bax^−/−^/Bak^−/−^* MEFs treated with 400 µM DETA-NO for 0, 8, 16 and 24 hours (A). MCL-1 degradation was measured in *Bax^−/−^/Bak^−/−^* MEFs pre-treated with or without 20 µM MG132 followed by 0 or 400 µM DETA-NO for 24 hours (B). MCL-1 protein expression was measured in *Jnk1^−/−^* (C), *Ask1^−/−^* (D) MEFs treated with 400 µM DETA-NO for the indicated time points.

JNK has been reported to inactivate the anti-apoptotic BCL-2 protein, MCL-1. We examined whether the ASK1-JNK1 axis negates MCL-1 as a mechanism for NO-dependent cell death. A widely accepted mechanism for MCL-1 negation following a death stimulus involves proteosomal degradation of the MCL-1 protein [34], [35]. To determine if MCL-1 turnover is affected by NO treatment upstream of BAX and BAK, we treated *Bax^−/−^/Bak^−/−^* MEFs with DETA-NO and measured MCL-1 protein expression. NO induced a decrease in MCL-1 protein levels upstream of BAX and BAK (Figure 9A). To determine if MCL-1 turnover is the result of proteosomal degradation, *Bax^−/−^/Bak^−/−^* MEFs were treated with DETA-NO in the presence of a the proteosomal inhibitor MG-132. The NO-induced decrease in MCL-1 protein was inhibited in cells treated with the proteosomal inhibitor (Figure 9B). To determine if ASK1 or JNK1 mediates NO-induced MCL-1 degradation, *Jnk1^−/−^* MEFs or *Ask1^−/−^* MEFs were exposed to DETA-NO. MCL-1 protein levels remained stable in *Jnk1^−/−^* MEFs and in *Ask1^−/−^* MEFs exposed to DETA-NO (Figure 9C and 9D). These results indicate that NO activates ASK1-JNK1 axis to initiate the degradation of MCL-1.

### Nitric oxide-induced MCL-1 degradation occurs through a non-canonical pathway

**Figure 10:**
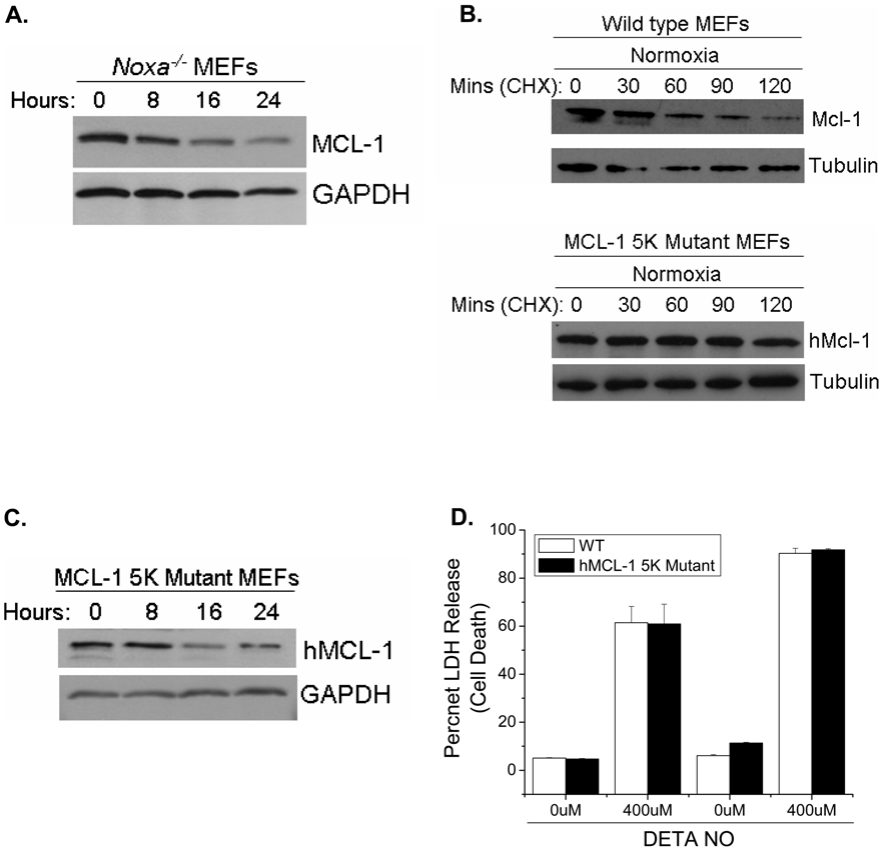
Nitric oxide-induced MCL-1 degradation occurs through a non-canonical pathway. MCL-1 protein expression was measured in *Noxa^−/−^* MEFs treated with 400 µM DETA-NO for 0, 8, 16 and 24 hours. Wild type and MCL-1 5K mutant MEFs were exposed to normoxia for 14 hours and subsequently treated with 5 µg/mL cycloheximide to inhibit protein translation. Cell lysates were collected at 30 minute intervals for 2 hours and MCL-1 expression was analyzed by Western blot. Tubulin served as a loading control (B). MCL-1 5K mutant MEFs were treated with 400 µM DETA-NO for 0, 8, 16 and 24 hours (C). Wild type and MCL-1 5K mutant MEFs were treated with 0 and 400 µM DETA-NO for 24 and 48 hours. Cell death was measured by percent LDH release (D).

The canonical pathway for MCL-1 degradation involves the activation of the BH3-only protein NOXA, which binds MCL-1 and induces its degradation by the proteosome [14], [36]. To examine NOXA's involvement in NO-dependent MCL-1 degradation, *Noxa^−/−^* MEFs were treated with DETA-NO and MCL-1 protein expression was assessed. MCL-1 is degraded in the absence of NOXA following NO treatment (Figure 10A). The E3 ligase, Mule, polyubiquitinates MCL-1 at five lysine residues (5, 40, 136, 194 and 197) to initiate proteosomal degradation [37]. Mutation of these five critical residues markedly increases the half-life of MCL-1 (Figure 10B). To determine if these five residues are required for NO-induced MCL-1 degradation, MEFs expressing MCL-1 with mutations at all five of these critical residues (MCL-1 5K mutant MEFs) were exposed to DETA-NO. Surprisingly, the 5K mutant MCL-1 is degraded following treatment with NO (Figure 10C). Additionally, MEFs expressing the 5K mutant die in response to NO (Figure 10D). These data indicate that NO induces degradation of MCL-1 through a non-canonical pathway.

### Nitric oxide-induced cell death is not initiated by ROS generation or peroxynitrite formation

**Figure 11:**
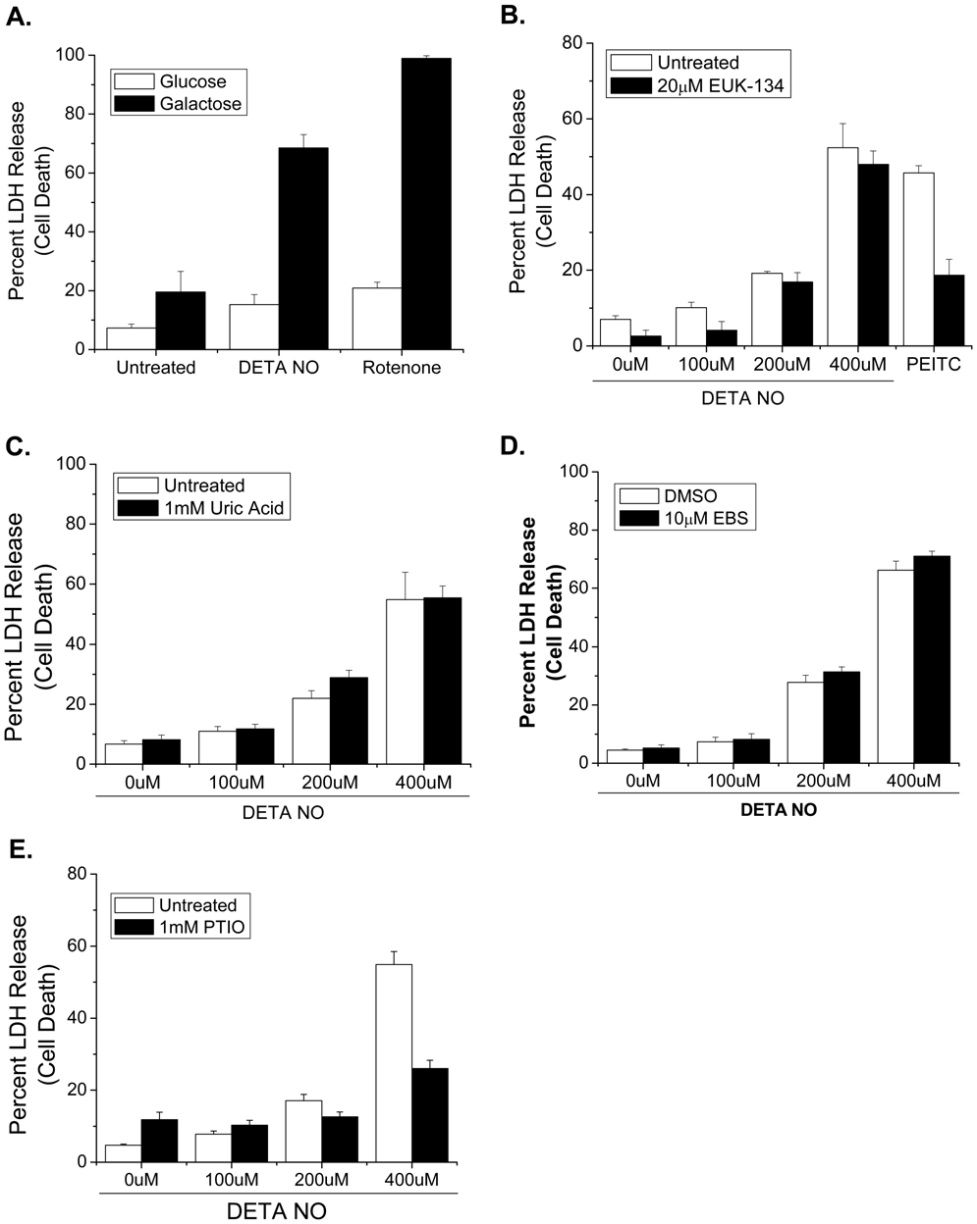
NO-induced apoptosis is not due to ROS or peroxynitrite generation. *Bax^−/−^/Bak^−/−^* MEFs adapted to glucose or galactose were treated with 400 µM DETA-NO or 10 µM rotenone for 48 hours and cell death was measured by percent LDH release (A). Wild type MEFs were pre-treated with the ROS inhibitor EUK-134 (20 µM) followed by 0, 100, 200 and 400 µM DETA-NO for 24 hours and cell death was measured by LDH release (B). PEITC is known to generate endogenous ROS and was used as a positive control. Wild type MEFs were pre-treated with the peroxynitrite scavengers, uric acid (1 mM) and ebselen (10 µM) followed by 0, 100, 200 and 400 µM DETA-NO for 24 hours and cell death was measured by percent LDH release (C and D). Wild type MEFs were pre-treated with the nitric oxide scavenger PTIO (1 mM) followed by 0, 100, 200 and 400 µM DETA-NO for 24 hours and cell death was measured by percent LDH release (E).

A mechanism by which NO could activate the ASK1-JNK1 axis to initiate BAX/BAK-dependent cell death is through ROS generation. ASK1 and JNK1 are both known to be activated by oxidative stress [38]–[40]. NO can increase mitochondrial oxidative stress by inhibiting cytochrome c oxidase [41]. This could cause the upstream electron transport chain to exist in a more reduced state and enhance superoxide production [5]. To functionally test whether NO inhibits respiration, we cultured *Bax^−/−^/Bak^−/−^* MEFs in media containing galactose instead of glucose. Supplementing glucose for galactose reduces glycolysis and forces cells to rely heavily on oxidative phosphorylation for ATP generation and survival. *Bax^−/−^/Bak^−/−^* MEFs are protected against DETA-NO in media containing glucose (Figure 1A–D). Thus, if NO inhibits mitochondrial respiration, *Bax^−/−^/Bak^−/−^* MEFs cultured in galactose would die since they cannot utilize glycolysis to generate ATP for survival. Indeed, NO, like the complex I inhibitor rotenone, induces cell death in *Bax^−/−^/Bak^−/−^* MEFs when cultured in galactose (Figure 11A). These results indicate that NO inhibits mitochondrial respiration. Surprisingly, EUK-134, which is a SOD/catalase mimetic and reduces ROS, did not protect cells from DETA-NO (Figure 11B). PEITC depletes glutathione and generates an increase in intracellular ROS [42]. EUK-134 protected MEFs from PEITC-induced cell death (Figure 11B). Additionally, wild type MEFs pre-treated with the peroxynitrite scavengers, uric acid or ebselen, also died at rates similar to controls (Figure 11C and 11D). By contrast, the NO scavenger PTIO decreased DETA-NO-induced cell death (Figure 11E). These results indicate that NO-induced activation of BAX/BAK-dependent cell death is independent of peroxynitrite formation and ROS generation.

## Discussion

Previous studies have demonstrated that the overexpression of anti-apoptotic BCL-2 family members prevents NO-induced cell death, suggesting that the NO induces cell death through the activation of the intrinsic apoptotic pathway [16]. BAX and BAK are integral members of the intrinsic pathway. Activation of these proteins results in cytochrome c release from the mitochondria and activation of Caspase-9. Our data show that BAX and BAK are activated by NO and that cytochrome c is released from the mitochondria. The combined loss of BAX and BAK, or the individual loss of Caspase-9, completely prevents NO-induced cell death indicating that these proteins are required for this pathway.

Activation of BAX and BAK is dependent on interactions between anti-apoptotic and pro-apoptotic BCL-2 proteins. There are currently two models that attempt to explain how these interactions orchestrate BAX and BAK activation. Despite differences in these models, both agree that BH3-only proteins are major upstream regulators of BAX and BAK activity. Both models agree that BIM, BID and PUMA are potent activators of apoptosis but through distinct mechanisms. The indirect activation model views these proteins as potent because they are able to bind and inhibit all of the anti-apoptotic BCL-2 proteins, whereas other BH3-only proteins have selective partners [43]. In this model, the negation of BCL-2 proteins is sufficient to induce BAX and BAK activation. The indirect activation model proposes that anti-apoptotic BCL-2 members negate the activity of BAX or BAK in healthy cells. In response to a death stimulus, BH3-only proteins such as BIM bind to the anti-apoptotics and displace them from BAX and BAK. By contrast, the direct activation model proposes that BIM, BID and PUMA are potent because they can directly activate BAX and BAK [15], [44].

In the present study, we find that cells collectively deficient in *Bim* and *Puma* with reduced *Bid* expression still die in response to NO. We speculate that a BH3-only protein other than BIM, BID, PUMA, BAD or NOXA negates the anti-apoptotics in response to NO or that NO promotes binding of multiple proteins to anti-apoptotic BCL-2 members, negating their activity.

A major mechanism for negating anti-apoptotic BCL-2 protein is through degradation. For example, MCL-1 is degraded in response to a variety of death stimuli including DNA damage, cytokine withdrawal, and anoxia [20], [34], [35], [45], [46]. NO induces proteosomal degradation of MCL-1 even in the absence of BAX and BAK, suggesting this is an early event in the pathway. Previous studies indicate that the BH3-only protein NOXA stimulates MCL-1 degradation upon exposure to a death stimulus. Furthermore, MCL-1 protein is ubiquitinated at five critical lysine residues that are required for degradation. Surprisingly, NO causes MCL-1 degradation in the absence of NOXA, and a mutant form of MCL-1, that can not be ubiquitinated at the five critical lysine residues, is also degraded. Collectively, these data indicate that MCL-1 is degraded by a non-canonical mechanism that does not involve NOXA or ubiquitinylation of MCL-1 at five specific lysine residues.

The signaling pathways that negate anti-apoptotic BCL-2 proteins to allow BAX and BAK activation are not fully understood. The JNK and p38 MAPK family members have been implicated as death kinases. Specifically, JNK1 but not JNK2, is implicated in the induction of cell death [21]. JNK can phosphorylate BIM or BID to induce BAX/BAK dependent cell death [29], [47]. JNK can also phosphorylate and inactivate MCL-1 [48], [49]. Previous studies have shown that NO can activate JNK and/or p38 MAPK, and that activation of these signaling pathways can induce cell death [50]. Our data indicate that p38α and JNK2 are dispensable in NO induced cell death, but *Jnk1* null cells are markedly protected from NO-induced cell death. Furthermore, NO does not induce MCL-1 protein degradation in the absence of *Jnk1*. These results indicate that in the absence of *Jnk1*, cell survival is due to the maintenance of MCL-1.

ASK1 activates the JNK signaling pathway under conditions of high oxidative stress. ASK1 is associated with the reduction/oxidation (redox)-regulatory protein, thioredoxin (Trx) [51]. ROS oxidize cysteine residues of the Trx protein causing it to dissociate from ASK1 resulting in its activation through autophosphorylation. Indeed, MCL-1 protein levels were maintained in *Ask1* deficient cells and these cells did not undergo cell death. However, we found no evidence that the NO-induced cell death pathway was dependent on oxidative stress. This was surprising since NO can inhibit the mitochondrial respiratory chain and increase oxidative stress [41]. In our experiments, NO was able to block oxidative phosphorylation. We speculate that NO might cause direct *S*-nitrosylation of the cysteine residues of Trx thereby liberating ASK1 to induce cell death.

In summary, our data reveal that NO negates anti-apoptotic BCL-2 family members, in part through the activation of the ASK1-JNK1 pathway, which leads to BAX/BAK-dependent cell death. These findings have implications for the role of inflammation in cancer progression. Recent data indicate that inflammation can exacerbate cancer progression. MCL-1 and BCL-X_L_ are up-regulated in many types of cancers. Activated macrophages, which release high levels of NO during inflammation, are capable of destroying neoplastic cells. However, if BCL-2 proteins are upregulated in tumor cell this would prevent NO-dependent cell death resulting tumor progression. This would further select tumor cells that have elevated levels of anti-apoptotic BCL-2 proteins and make them resistant to chemotherapy or radiotherapy.

## Methods

### Cell lines and cell culture

*Bax^−/−^/Bak^−/−^*, *Caspase-9^−/−^*, *Bim^−/−^*, *Bad^−/−^*, *Bid^−/−^*, *Puma* ^−/−^, *Noxa* ^−/−^, *Jnk1* ^−/−^,*Jnk2* ^−/−^, *Ask1* ^−/−^, p38^−/−^ MEFs and their wild type controls were cultured as previously reported [20]–[22]. *Bim^−/−^/Puma^−/−^* MEFs and their wild type controls and PT67 were cultured in Dulbecco's modified essential medium (DMEM) with 4.5 g/liter glucose, l-glutamine, and sodium pyruvate (Invitrogen). Media was supplemented with 10% heat-inactivated fetal bovine serum (FBS) (Invitrogen), 100 U/ml penicillin/100 µg/ml streptomycin (P/S) (Cellgro), 20 mM HEPES (Cellgro) and 1X NEAA (Invitrogen). MCL-1 5K mutant MEFs and 293FT were cultured in the above media supplemented with G418. *Bim^−/−^/Puma^−/−^* MEFs were transformed with dominant negative p53. Galactose media was prepared as follows, DMEM without glucose (GIBCO), 10% FBS, 20 mM galactose, P/S, HEPES.

### Measurement of cell death and apoptosis

Cell death was measured using the Cytotoxicity Detection Kit (Roche Applied Science) according to manufacturer's protocol. The kit is a colormetric assay based on the measurement of lactate dehydrogenase (LDH) released by dead cells. Percent cell death is determined by the amount of LDH measured in the medium divided by the amount of LDH after addition of 1% Triton-X 100, which kills all the cells.

Apoptosis was measured using the Annexin V-FITC Apoptosis Detection Kit (BD Pharmingen) according to manufacturer's protocol. After treatment, cells were washed twice with cold PBS and resuspended in 1X binding buffer at a concentration of 1×10^6^ cells per milliliter. Annexin-V-FITC (excitation: 488 nm, emission: 525) was added to the cells and analyzed by flow cytometry.

### Measurement of BAX and BAK activation

BAX and BAK activation was determined as previously published [23]. Briefly, adherent and non-adherent cells were collected in 1X Cell Dissociation Solution Non-enzymatic (Sigma). Cells were fixed in 0.25% paraformaldehyde for 1 minute then washed with phosphate-buffered saline (PBS). Cells were incubated with anti-Bax antibody (BD Pharmingen clone 6A7) or anti-Bak antibody (Calbiochem) for 30 minutes at a concentration of 1∶50 in PBS containing 100 µg/mL digitonin (Sigma). Cells were washed with PBS then incubated in FITC-conjugated rat anti-mouse immunoglobulin (BD Pharmingen clone A85-1) at a concentration of 1∶50 in PBS containing 100 µg/mL digitonin. Cells were washed in PBS and analyzed by flow cytometry.

### Measurement of cytochrome c release

Cells were collected in mitochondrial isolation buffer (250 mM Sucrose, 10 mM Tris-HCl pH 7.4, 0.1 mM ethylene glycol tetraacetic acid (EGTA)) and homongenized on ice using a Dounce homogenizer. Cells were then expelled through a 27-guage needle 10 times. Samples were centrifuged at 1000rpm for 10 minutes. Subsequently, the supernatants were centrifuged at 15,000rpm for 20 minutes to pellet the mitochondria. The mitochondrial pellet was solubilized in mitochondrial isolation buffer containing 10X lysis buffer.

### Antibodies and Western blot analysis

Phospho-JNK, JNK, phospho-cJun and cJun antibodies were ordered from Cell Signaling. Additionally, antibodies used for Western blot analysis include: Mcl-1 (Rockland), Bid (R&D Systems), Bim (BD Pharmingen), Cytochrome c (MitoSciences), β-actin (Sigma), Tubulin (Sigma), and GAPDH (Santa Cruz). Briefly, cell lysates were collected in 1X Cell Lysis Buffer (20 mM Tris-HCl (pH 7.5), 150 mM NaCl, 1 mM Na_2_EDTA, 1 mM EGTA, 1% Triton, 2.5 mM sodium pyrophosphate, 1 mM beta-glycerophosphate, 1 mM Na_3_VO_4_, 1 µg/ml leupeptin) (Cell Signaling) and amount of total protein was determined by Bradford Assay. Lysates were separated on a 10% Tris-HCl Polyacrilimide Gel (BioRad) and transferred to a nitrocellulose membrane. Membranes were incubated in blocking buffer (1X TBS, 0.1% Tween-20 with 5% nonfat dry milk) for 1 hour followed by primary antibody incubation at 4°C overnight. The following day, membranes were washed and then incubated in HRP linked anti-mouse or anti-rabbit (Cell Signaling) for 1 hour. SuperSignal chemiluminescent substrate (Pierce) was used to detect protein levels.

### Lentiviral production and generation of stable cell lines

The pLKO.1 vector was used to express shRNA targeting Bid. Constructs were ordered from Open Biosystems with the following hairpin sequences. Sequence #1, 5′

ccgggctccttcaaccaaggaagaactcgacttccttggttgaaggagcttttt, Sequence #2, 5′

ccggccacacgactgtaactttatctcgagataaagttgacagtcggtggttttt. The non-silencing shRNA was ordered from Addgene (plasmid 1864), 5′ cctaaggttaagtcgccctcgctctagcgagggcgacttaaccttagg 3′. Stable cell lines were generated using lentiviral infection using the 293FT packaging cell line and puromycin selection.

### BCL-X_L_ overexpression

The Gateway® Lentiviral Expression Kit (Invitrogen) was used to overexpress FLAG® tagged BCL-XL. Flag-BCL-XL was cloned into pDONR221 by BP recombination and then into pLenti6/V5-DEST by LR recombination. Stable cell lines were generated by transfecting the 293FT packaging cell line.

### MCL-1 5K mutant construct generation and expression

The pLNCX vector containing human MCL-1 was a kind gift from Dr. Aly Karsan. The MCL-1 5K mutant was made using Site-Directed Mutagenesis (Stratagene) following manufacturer's instructions. Lysines at positions 5, 40, 136, 194 and 197 of MCL-1 were changed to arginines using the following primers: K5R, forward 5′ ggcaatgtttggcctcagaagaaacgcggtaatcgg 3′ and reverse 5′ ccgattaccgcgtttcttctgaggccaaacattgcc 3′, K40R: forward 5′ cgacttttggctacggagagggaggcctcg 3′ and reverse 5′ cgaggcctccctctccgtagccaaaagtcg 3′,

K136R: forward 5′ cggagcctctcgggaggcggccgg 3′ and reverse - 5′ ccggccgcctcccgagaggctccg 3′, K194/197R forward 5′ ggccaccggcgccagggacacagagccaatg 3′ and reverse 5′ cattggctctgtgtccctggcgccggtggcc 3′. Mutations were confirmed by DNA sequencing. Stable cell lines were generated by transfecting the PT67 packaging cell line using Mirus *Trans* IT Transfection reagent (Mirus Bio Corporation) according to the manufacturer's protocol. Cell lines were selected with G418 (Sigma).

### Other Reagents

The following reagents were used: (Z)-1-[*N*-(2-Aminoethyl)-*N*-(2-ammonioethyl)amino]diazen-1-ium-1,2-diolate (DETA-NO, Alexis® Biochemicals), Z-Leu-Leu-Leu-aldehyde (MG-132, 20 µM, Calbiochem), SP600125 (40 µM, A.G. Scientific, Inc.), Ebselen (10 µM, A.G. Scientific), chloro[[2,2'-[1,2-ethanediylbis[(nitrilo-.kappa.N)methylidyne]]

bis[6-methoxyphenolato-.kappa.O]]]-manganese (EUK-134, 20 µM, Cayman Chemical), uric acid (1 mM, MP Biochemicals), 2-Phenyl-4,4,5,5-tetramethylimidazoline-1-oxyl 3-oxide (PTIO, 1 µM, Sigma), phenethyl isothiocyanate (PEITC, 20 µM, Sigma), UO126 (10 µM, Sigma), and rotenone (10 µM, Sigma).
